# Effect of Clark's twin-block appliance (CTB) and non-extraction fixed mechano-therapy on the pharyngeal dimensions of growing children

**DOI:** 10.1590/2177-6709.20.6.082-088.oar

**Published:** 2015

**Authors:** Batool Ali, Attiya Shaikh, Mubassar Fida

**Affiliations:** 1 Resident in Orthodontics, Aga Khan University Hospital, Department of Surgery, Section of Dentistry, Karachi, Pakistan; 2 Assistant professor, Aga Khan University Hospital, Residency program, Department of Surgery, Section of Dentistry, Karachi, Pakistan; 3 Associate professor, Program Director, Aga Khan University Hospital, Residency program, Department of Surgery, Section of Dentistry, Karachi, Pakistan

**Keywords:** Functional appliance, Twin-block, Pharyngeal passage, Mandibular retrognathia

## Abstract

**Introduction:**

Narrow airway dimensions due to mandibular deficiency can predispose an individual to severe respiratory distress. Hence, treatment with mandibular advancement devices at an early age might help improving the pharyngeal passage and reduce the risk of respiratory difficulties. Therefore, the aim of the current study was to evaluate the mean changes in the pharyngeal dimensions of children with mandibular deficiency treated with Clark's twin-block appliance (CTB) followed by fixed orthodontic treatment.

**Methods:**

Orthodontic records of 42 children with mandibular deficiency were selected. Records comprised three lateral cephalograms taken at the start of CTB treatment, after CTB removal and at the end of fixed appliance treatment, and were compared with 32 controls from the Bolton-Brush study. Friedman test was used to compare pre-treatment, mid-treatment and post-treatment pharyngeal dimensions. Wilcoxon signed rank test was used to compare the airway between pre-treatment and post follow-up controls. Mann-Whitney U test was applied to compare the mean changes in pharyngeal dimensions between treatment group and controls from T_2_ to T_0_. Post-hoc Dunnet T3 test was used for multiple comparisons of treatment outcomes after CTB and fixed appliances, taking a *p*-value of ≤ 0.05 as statistically significant.

**Results:**

Superior pharyngeal space (*p* < 0.001) and upper airway thickness (*p* = 0.035) were significantly increased after CTB, and the change in superior pharyngeal space remained stable after fixed mechano-therapy.

**Conclusion:**

CTB can have a positive effect in improving pharyngeal space and the resultant increase in airway remains stable on an average of two and a half years.

## Resumo

**Introdução:**

a redução nas dimensões das vias aéreas causada pela deficiência mandibular pode predispor um indivíduo a dificuldades respiratórias severas. Assim, o tratamento com aparelhos de avanço mandibular na infância pode contribuir para melhorar a via aérea faríngea e reduzir o risco de problemas respiratórios.

**Objetivo:**

o objetivo do presente estudo foi avaliar as alterações médias nas dimensões da faringe de crianças com deficiência mandibular tratada com o aparelho Twin Block (TBC) seguido pelo tratamento ortodôntico fixo.

**Métodos:**

a documentação ortodôntica de 42 crianças com deficiência mandibular, consistindo de três telerradiografias de perfil - tiradas ao início do tratamento com TBC (T_0_), após a remoção do aparelho (T_1_) e ao final do tratamento ortodôntico fixo (T_2_) - foi selecionada e comparada à de 32 crianças controle do estudo Bolton-Brush. O teste de Friedman foi utilizado para comparar as dimensões da faringe antes, durante e após o tratamento. O teste de postos de Wilcoxon foi utilizado para comparar as vias aéreas antes do tratamento e depois do acompanhamento das crianças controle. O teste U de Mann-Whitney foi empregado para comparar as alterações médias nas dimensões da faringe entre o grupo tratado e as crianças controle, de T_0_ a T_2_. O teste T3 de Dunnett foi utilizado como *post-hoc*para realizar comparações múltiplas dos resultados do tratamento após o uso do TBC e dos aparelhos fixos, considerando-se como estatisticamente significativo um valor de *p* ≤ 0,05.

**Resultados:**

o espaço faríngeo superior (*p* < 0,001) e a espessura das vias aéreas superiores (*p* = 0,035) aumentaram significativamente após o uso do TBC, e a alteração no espaço faríngeo superior permaneceu estável após a mecanoterapia fixa.

**Conclusão:**

o TBC pode produzir um efeito positivo no espaço faríngeo, e aumento resultante nas vias aéreas permanece estável, em média, por dois anos e meio.

## INTRODUCTION

The anatomy and function of nasopharyngeal airway is directly associated with craniofacial development. The growth of the cranial base, along with an increase in the nasopharyngeal dimensions, results in a downward and forward displacement of the midface and its associated structures.^1^Various studies have reported that the abnormal position and atypical growth pattern of dental and craniofacial structures can influence pharyngeal dimensions.^2^^,^ 3 ^,^ 4 Similarly, physiological impairment of the nasopharynx due to adenoidal hypertrophy or nasal stenosis can result in growth disturbances leading to adenoid facies (long face syndrome) which is associated with mouth breathing and an altered cranio-cervical posture.^1^^,^ 2 Anatomical and physiological factors, such as short mandible, increased size of the tongue and soft palate, posteriorly postured tongue and vertical growth discrepancy may also play a role in narrowing the airway.^5^^,^ 6 ^,^ 7 Mandibular retrognathism has been considered one of the most important risk factors in children and adolescents suffering from sleep disordered breathing or Obstructive Sleep Apnea (OSA).^8,9^

OSA is a clinical disorder characterized by recurring episodes of upper airway obstruction leading to reduced or absent airflow through the nasal or oral cavity. Upper airway resistance is remarkably increased by macroglossia, hypertrophic soft palate impinging the hypo-pharyngeal space along with supine posture and hypotonic airway muscles.^10^^,^ 11 Additionally, anteroposterior discrepancy of the maxilla and mandible due to a micrognathic or retrognathic mandible can lead to significant constriction of the retropalatal and retroglossal areas, resulting in critical narrowing of airway.^12^^,^ 13 Hence, relieving constriction and increasing the pharyngeal dimensions at these sites are among the primary goals of OSA treatment.

Mandibular deficiency being one of the common causes of respiratory distress is also a clinical presentation in subjects with skeletal Class II malocclusion. Subjects with respiratory difficulties might present with an underlying Class II malocclusion and vice versa. Banabilh et al,^14^ in their study conducted on Malay subjects with OSA, reported the frequency of convex facial profile and Class II malocclusion as 71.7% and 51.7%, respectively. Similarly, another study reported a 26.5% incidence of OSA in Class II subjects.^15^

Class II malocclusion due to deficient mandible, if diagnosed at an early age, can be treated with functional appliances. Similar oral appliances are also used in adult OSA patients to prevent upper airway collapse during sleep.^16^^,^ 17 Orthodontic treatment with such appliances used to bring the lower jaw forward prevents the posterior relocation of the tongue and improves pharyngeal airway passage along with enhancing facial esthetics.^18 ^Various studies have been conducted to evaluate the effects of different mandibular advancement devices, such as Harvold activator, modified bionator and Clark's twin-block (CTB), on mandibular growth and the changes occurring in pharyngeal dimensions of growing skeletal Class II children,^18^^-^ 21 but very few studies have evaluated the long-term effects achieved by these oral appliances. To our knowledge, only few studies have reported whether the increase in airway size is solely due to the functional appliance or is a combination of functional appliance and fixed mechano-therapy, and whether the positive effects achieved with these functional appliances last even after the completion of fixed orthodontic treatment.

Hence, the aim of our study was to evaluate the mean changes in pharyngeal dimensions in growing children with skeletal Class II malocclusion treated with CTB followed by non-extraction fixed mechano-therapy.

## MATERIAL AND METHODS

A retrospective study was conducted on 42 children (21 males, 21 females), with a mean age of 10.4 ± 1.27 years, treated with CTB associated with fixed orthodontic appliances. Sample size was calculated keeping a = 0.05, power of study (b) as 80 % and using the findings of Jena et al^18^ who reported a mean difference of 2.12 ± 0.67 mm for the middle pharyngeal space between treatment and control groups. Subjects having skeletal Class II malocclusion (ANB > 4°) due to mandibular deficiency (SNB < 78°), normal vertical growth pattern (SN to Go-Gn angle = 32 ± 4°), and bilateral Angle's Class II malocclusion, compliantly treated with CTB, were included in the study. None of the subjects in the study group had undergone any pre-functional orthodontic treatment. Subjects with respiratory problems, obvious nasopharyngeal obstructions, upper airway surgeries, craniofacial anomalies and syndromes, trauma, history of previous orthodontic treatment or absence of acceptable quality of radiographs at all three treatment time intervals were excluded from the study.

The total treatment duration was 36.5 ± 6.1 months with an average of 8.14 ± 2.9 months of CTB treatment followed by 28.3 ± 6.5 months of non-extraction fixed mechano-therapy. The initial lateral cephalometric radiographs of treatment subjects were taken prior to the start of treatment (T_0_). The mid-treatment radiographs were taken after the removal of the CTB appliance (T_1_) and post-treatment lateral cephalometric radiographs were taken after completion of the non-extraction fixed mechano-therapy (T_2_). Subjects in the treatment group were instructed to wear the appliance 24 hours per day, removing it only at meal times and during brushing. All the appliances were constructed with an expansion screw which was activated by means of the slow expansion protocol of one turn every alternate day (0.25 mm/turn). The construction bite of the appliance was recorded with a vertical opening of 2-3 mm between upper and lower incisors and sagittally advancing the mandible to an edge-to-edge incisor relationship. To maintain the vertical dimension, the inter-occlusal acrylic was trimmed incrementally at each visit. CTB treatment was followed by non-extraction fixed mechano-therapy with pre-adjusted Edgewise appliances (Roth prescription, 0.022 x 0.028-in slot). All subjects were treated by a single clinician following the same treatment protocol.

The control group consisted of 32 subjects (16 males, 16 females) taken from the Bolton-Brush study with no history of orthodontic treatment, and was matched in skeletal age (CVM III at initial radiographs), sex, and ANB angle with the experimental subjects. The first radiograph from the Bolton-Brush study (T_0_) was taken at an average age of 10.1 ± 0.78 years, while the second radiograph was taken after three years to match with the post-treatment readings of the study group. All treated and control subjects showed a circumpubertal stage of skeletal growth (CS 3 as reported by Baccetti et alz^19^) at T_0_.

In order to ensure a high degree of precision, pre-, mid and post-treatment lateral cephalograms were routinely taken in an erect position, with the FH plane being parallel to the ground, and teeth in centric occlusion. These radiographs were recorded with rigid head fixation and a 165-cm film-to-tube distance, using Orthoralix^TM^ 9200 (Gendex-KaVo, Milan, Italy). 

**Figure 1:**
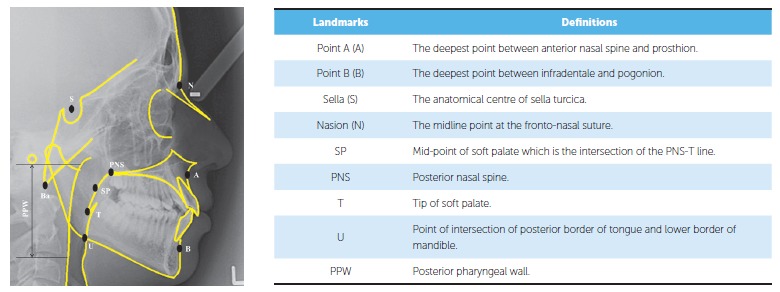
- Anatomical landmarks used for skeletal and pharyngeal analysis.

Cephalograms were traced manually with a 0.5-mm lead pencil, on acetate sheets on an illuminator, and landmarks were identified as seen in Figure 1. 

**Figure 2:**
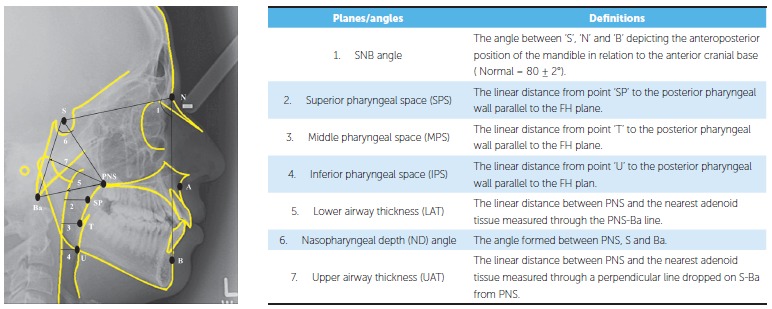
- Skeletal and pharyngeal measurements.

Linear and angular readings were measured with the help of a millimetric ruler and a protractor, respectively. Corrected values of linear measurements were recorded to eliminate a magnification error of 10%. The linear and angular measurements used to evaluate the pharyngeal airway and the relationship of the mandible with the cranial base, as well as definitions of the cephalometric planes and angles used in this study, are shown in Figure 2. Measurements of 30 randomly selected lateral cephalograms were repeated by the main investigator four weeks after initial analysis. The first and second readings were compared by means of the intraclass correlation coefficient (ICC) which showed greater than 0.90 intraexaminer reliability for all variables assessed. 

Statistical analyses for the collected data were performed using SPSS software for Windows (version 20.0; SPSS, Chicago, III). For linear variables, means and standard deviations of measurements were computed at three different intervals. Shapiro-Wilk test was used to check the normality of measurements which showed a non-normal distribution of data. Friedman test was used to compare pre-treatment (T_0_), mid-treatment (T_1_) and post-treatment (T_2_) pharyngeal dimensions. Post-hoc Dunnet T3 test was used for multiple comparisons of treatment outcomes after CTB and fixed appliances. The mean changes within the control group (pre-treatment and post follow up) were determined by Wilcoxon signed rank test; whereas the mean differences between treatment and control groups were compared by Mann-Whitney U test. A *p*-value of ≤ 0.05 was assigned as statistically significant for all test results.

## RESULTS

Pre-treatment pharyngeal dimensions were compared between males and females, and no significant differences were found between them; hence, two groups were further statistically analyzed as one to increase the power of the study (Table 1). 

**Table 1:** - Comparison of changes in pharyngeal dimensions between males and females before treatment.

**Variables**	**Treatment group**		**Control group**	***p*-value**		
	**Males (n = 21)**	**Females (n= 21)**	***p*-value**	**Males (n = 16)**	**Females (n = 16)**	
SPS (mm)	13.23 ± 2.27	13.04 ± 2.16	0.82	12.40 ± 2.28	12.93 ± 2.29	0.56
MPS (mm)	11.04 ±2.76	11.50 ± 4.18	0.07	10.37 ± 2.02	10.54 ± 1.89	0.36
IPS (mm)	11.42 ± 3.35	10.92 ± 2.58	0.81	8.16 ± 1.58	8.91 ± 2.66	0.64
LAT (mm)	26.38 ± 5.47	24.80 ± 3.17	0.34	30.31 ± 2.75	32.53 ± 4.97	0.06
ND (degree)	59.09 ± 6.87	59.04 ± 5.23	0.61	65.12 ± 4.28	66.43 ± 3.94	0.42
UAT (mm)	34.57 ± 5.27	33.04 ± 2.99	0.07	41.09 ± 3.57	42.87 ± 5.26	0.16

Mann-Whitney U test.

The skeletal and pharyngeal dimensions in treatment and control groups are described in Table 2. Friedman test comparing the pharyngeal changes after CTB and fixed appliances at three different intervals (T_0_, T_1_ and T_2_) showed a highly significant increase in mandibular position (*p* < 0.001), superior pharyngeal space (*p* < 0.001) and upper airway thickness (*p*< 0.001) at the end of orthodontic treatment. Individual paired comparisons of treatment outcomes after CTB and fixed appliance therapy showed a significant increase in superior pharyngeal space (*p* = 0.009) from T_0_to T_1_, and the change remained stable after the completion of fixed appliance treatment, i.e, from T_0_to T_2_(*p* = 0.004). However, significant change in upper airway thickness (*p* = 0.036) was observed only from T_0_to T_2_, which indicates that the change was due to a combination of functional and fixed appliance treatment (Table 3).

**Table 2:** - Changes in pharyngeal dimensions between treatment and control groups.

**Skeletal and pharyngeal dimensions**	**Treatment Group ¥**	**Control Group &**					
	**( n = 42 )**	**( n = 32)**					
	**T_0_**	**T_1_**	**T_2_**	***p*-value**	**T_0_**	**T_2_**	***p*-value**
	**Mean ± SD**	**Mean ± SD**	**Mean ± SD**		**Mean ± SD**	**Mean ± SD**	
SNB (degree)	74.57 ± 3.12	75.80 ± 3.50	76.42 ± 3.61	< 0.001**	72.83 ± 1.89	73.19 ± 1.59	0.11
SPS (mm)	13.14 ± 2.19	15.07 ± 3.43	14.97 ± 2.78	< 0.001**	12.67 ± 2.26	12.42 ± 2.41	0.57
MPS (mm)	10.36 ± 3.63	10.97 ± 2.68	11.08 ± 2.81	0.032	9.88 ± 2.13	10.12 ± 2.43	0.35
IPS (mm)	11.17 ± 2.96	11.78 ± 3.36	11.72 ± 3.04	0.146	8.54 ± 2.19	8.88 ± 2.45	0.27
LAT (mm)	25.59 ± 4.71	25.57 ± 4.60	26.45 ± 4.81	0.087	29.92 ± 5.24	31.18 ± 4.57	0.04*
ND (degree)	59.07 ± 6.03	58.14 ± 5.28	58.52 ± 5.42	0.489	64.53 ± 4.73	64.71± 4.08	0.72
UAT (mm)	32.92 ± 4.53	33.88 ± 4.23	35.50 ± 4.67	< 0.001*	41.98 ± 4.51	43.75 ± 4.44	< 0.001**

*p ≤ 0.05; ** *p* ≤ 0.001; ¥ Friedman test; & Wilcoxon signed rank test.

**Table 3:** - Changes in pharyngeal dimensions at different treatment intervals with CTB and fixed appliance mechano-therapy.

**Variables**	**T_0_ - T_1_**	**T_1_ - T_2_**	**T_0_ - T_2_**
	**(*p*)**	**(*p*)**	**(*p*)**
SPS	0.009*	0.998	0.004*
MPS	0.766	0.997	0.678
IPS	0.762	1.000	0.788
LAT	1.000	0.775	0.795
ND	0.837	0.983	0.961
UAT	0.687	0.269	0.036*

N = 42; *p ≤ 0.05; Post-hoc Dunnet T3 test.

The control group was analyzed by means of Wilcoxon signed rank test to see the effect on airway dimensions, and a statistically significant increase in upper airway thickness (*p* < 0.001) and lower airway thickness (*p* = 0.04) was observed (Table 2). 

The mean changes in pharyngeal airway dimensions from T_2_ to T_0_were compared by means of Mann-Whitney U test, as shown in Table 4. The superior pharyngeal space was significantly improved (*p* < 0.001) by 1.83 mm in the treatment group as compared to 0.25 mm in the controls. Upper airway thickness was significantly increased (*p* < 0.001) by 2.57 mm and 1.76 mm in the treatment and control groups, respectively. The improvement of upper airway thickness among treatment group subjects was significantly greater when compared to that of the controls (*p* = 0.03). 

**Table 4:** - Mean changes in pharyngeal dimensions between treatment and control group (T_0_ - T_2_).

**Variables**	**Treatment group**	**Control group**	***p*-value**
	**(n = 42)**	**(n = 32)**	
SPS(mm)	1.83 ± 2.73	-0.25 ± 2.14	< 0.001**
MPS (mm)	0.71 ± 3.45	0.24 ± 1.59	0.342
IPS (mm)	0.54 ± 2.24	0.34 ± 1.61	0.796
LAT (mm)	0.85 ± 4.16	1.26 ± 3.27	0.358
ND (degree)	-0.45 ± 3.25	1.87 ± 2.91	0.612
UAT (mm)	2.57 ± 1.46	1.76 ± 1.86	0.035*

*p ≤ 0.05; ** *p* ≤ 0.001; Mann-Whitney U test.

## DISCUSSION

Narrow airway dimensions secondary to anatomical or physiological constraints during craniofacial development can predispose an individual to severe respiratory distress. With advancing age, a decrease in oropharyngeal depth,^23^ an increase in the length and thickness of the soft palate,^24^ and clinical signs of obesity associated with subsequent soft tissue changes^23^ play a role in reducing oropharyngeal airway. Hence, treatment with mandibular advancement devices, functional appliances or surgical interventions at an early age can protect a child from long-term respiratory disturbances.^20^

According to the present study, the anteroposterior relationship of the mandible with the cranial base was significantly improved with CTB treatment, and this observation was similar to that found in previous studies.^18^^,^ 25 The results achieved in our study show that the change in pharyngeal dimensions after orthodontic intervention remain stable at least for a period of two and a half years. Since there was a significant increase in the SNB angle, these findings suggest that the sagittal discrepancy of the jaws is mainly corrected with anterior mandibular repositioning.

The current study highlights that the superior pharyngeal space is significantly increased after CTB treatment, and the increase in the superior pharyngeal space was maintained after two and a half years of fixed mechano-therapy. The results also revealed that not all changes in pharyngeal dimensions are affected by CTB treatment. The reported increase in the superior pharyngeal space is in concordance with multiple other studies;^18^^,^ 20 whereas few other studies reported an increase in superior and inferior airway dimensions, only.^26^^,^ 27 Similarly, the present study found an increase of 1.83 mm in the upper pharyngeal dimensions and no significant increase in the controls; whereas Han et al^28^ reported an increase of 2 mm in Class II subjects treated with Bionator and 0.8 mm improvement in upper airway in Class I controls. The heterogeneity in results might be due to racial differences and varying growth patterns of children, which acts as a confounder and could not be controlled in many studies due to ethical limitations.

In this study, we observed no significant changes in the inferior airway space and nasopharyngeal depth. In this regard, our results are comparable with those reported by Jena et al,^18^ Han et al^28^ and Erbas^29^ who evaluated the effects of CTB and MPA-IV, Bionator and Xbow on airway dimensions.

No significant effect on nasopharyngeal dimensions or thickness observed in our study might be due to the fact that the nasopharyngeal regions are associated with the change in the size of adenoids which are not affected by functional orthopedic treatment. However, in contrast to our observation, Vinoth et al^27^ found a significant increase in the above mentioned airway measurements. In addition to that, we also noticed a greater increase in upper airway thickness, as compared to the controls, but our results differ from the study conducted by Ghodke et al^30^ who observed that the twin-block appliance has no positive effect on the posterior pharyngeal wall thickness of Class II subjects at various upper airway regions.

It is interesting to note that the major changes seen were in upper airway size and thickness, although the effects of CTB are primarily related to the forward positioning of the mandible. The expansion achieved in the upper arch, along with forward mandibular repositioning, may aid forward relocation of the tongue and thus increase the posterior tongue space. Additionally, the growth of the oropharyngeal capsule, due to stretch and stimulation of the oropharyngeal muscles caused by mandibular advancement, can also play a role in altering superior airway dimensions.^19^

Two-dimensional lateral cephalograms were used to evaluate a three-dimensional airway space, which could not reveal the possible changes in the transverse dimension. However, reproducibility of pharyngeal dimensions on two-dimensional cephalograms is highly accurate and, due to an additional excessive radiation dose of the three-dimensional imaging techniques, lateral cephalograms remain a valuable diagnostic tool in the assessment of the airways.^11^ Furthermore, 3D imaging is not routinely used for orthodontic diagnostic and treatment purposes, as it adds to the cost of overall treatment.

Due to being a retrospective study design, the Body Mass Index (BMI) of subjects could not be recorded; hence, the confounding factor of obesity could not be ruled out. A control group sample to match with the CTB removal at T_1_ was not taken into account in the current study, as radiographs of an average of 8-9 month interval after pre-treatment were unavailable and radiographs of a 12-month interval could create potential bias in the results.

Thus, the findings of the study indicate that the CTB has a positive impact on airway and has the potential to alter superior pharyngeal dimensions by increasing the distance between the soft palate and the posterior pharyngeal wall. This is primarily achieved by altering tongue posture and redirecting the mandible forward, relieving airway constriction. Apart from this, the proposed concept of mandibular catch-up growth might aid in further resolving respiratory distress. The results of our study do not implicate that respiratory impairment of an individual will be corrected, as breathing is a complex phenomenon which cannot be treated solely by increasing the oropharyngeal dimensions.

## CONCLUSIONS

CTB has a marked effect in increasing superior pharyngeal space and upper airway thickness. Hence, this appliance can be used as a treatment modality not only to correct facial disharmony of children with a retrognathic mandible, but also to improve airway dimensions. Importantly, the resultant increase in the superior pharyngeal space with the twin-block appliance remains stable, according to the present study. However, long-term follow-up studies are needed to further explore the effectiveness and stability of the functional appliances in improving airway by controlling the confounder of growth. 
